# Biographical Sketch of Dr. Marshall Hall

**Published:** 1857-10

**Authors:** 


### EDITORIAL.

*Dr. Marshall Hall.*

In our last number we announced the death of this distinguished physician.

We are sure our readers will be interested with the following biographical sketch, taken from our exchanges:

Death, the most unsparing of tyrants, has exacted from the greatest physiologists of the age the last debt of nature. Slowly, surely, and relentlessly, disease has been undermining the earthly tabernacle of a mind which, for vast powers, high purposes, and indomitable energy, has found no superior in its native land in the present half-century. On Tuesday last, the 11th inst., Dr. MARSHALL HALL died at Brighton, aged 67 years.

It is impossible to record this melancholy event without feelings of the deepest sorrow. The loss is one which all must feel keenly who have a reverence for high endeavors, for earnest devotion to science, and for all the sterling qualities which can adorn a man. Science has lost the worthiest of her sons, medicine has lost a great master, and philosophy a great thinker. The clear and vivid intellect of this great man has steadily and successfully risen superior to the depressing influences of disease for the last fifteen years. Even during the present year, when confined to one room, his chamber has been a scene of intellectual activity. Physical debility, which robs most men of their powers of thinking and reasoning, had not dimmed the brightness of his wonderful mind. Clear and penetrating, and impelled by a wide philanthropy, the last contribution of Dr. Marshall Hall to science has been a pre-eminently useful one to the cause of humanity. It is thus that great men should die. There is a grandeur in such a life-end, to which the mere external grace of a falling Cæsar is not for one moment comparable.

Dr. Marshall Hall was born at Bashford, in Nottinghamshire, in the year 1799. His father was a manufacturer, and a man of no small capacity and information, and had the merit of being the first person to perceive the value of chlorine as a decolorizing agent, and applying it on a large scale. The gifts of intellect were bestowed with no sparing hand in this family. The father and two sons fully vindicated their claims to high intellectual endowments. But Dr. Marshall Hall has eclipsed his less brilliant relations. What in them was acumen and sagacity, was developed in him into genius. There was in him that rapid and far-searching intellectual vision which travels into regions far beyond the common ken of man, visible and appreciable only to the eagle glance of an almost prescient inquirer.

The history of such a man cannot fail to present numerous points of interest. The investigation of the rise and progress of a mind which has ever been foremost in the ranks of science, must afford many good and useful lessons. No fitful glare can be recognised in this life—no charlatanic attempt to pluck a crown of laurels which was not deserved; but a stern conscientious, and faithful continuance of patient scientific toil, and the solid reward of a vast reputation.

The first step in Dr. Marshall Hall's education was taken at Nottingham Academy, then conducted by the Rev. J. Blanchard. From this school he went to Newark, where he acquired some elementary and chemical knowledge. But the first salient point in the life of Dr. Marshall Hall was his matriculation at Edinburgh University, in the year 1809. For a vigorous and apt mind, no better school could then have been chosen. In the present day it is hardly possible to realize the enthusiasm which inspired Edinburgh at that time. There were giants in those days. Enthusiasm, indeed, is almost too tame a word. There was a furor, an excitement produced by the united influence of a complete galaxy of talent. It was impossible but that such men as Cullen, Home, Rutherford, Gregory, Hamilton, Bell, and Barclay should kindle in the ardent minds of a vast concourse of students a flame which should burn with answering brightness to their own. From the school of that time we know many great men have sprung. It is unnecessary to particularize names which are "familiar in our mouths as household words." In that genial atmosphere, then, did Marshall Hall first imbibe that enthusiastic love of science which has been his most marked characteristic. With youthful impetuosity he plunged into the study of chemistry. Not content with merely assimilating the accepted doctrines of the science, he boldly endeavored to push its boundaries farther. With wonderful power of generalization for so young a man, and with such small materials as then existed for the purpose, Dr. Marshall Hall pointed out that there was a grand distinction between all chemical bodies, which ruled their chemical affinities. He showed that this distinction was the presence or absence of oxygen. That oxygen compound combined with oxygen compounds, and compounds not containing oxygen with compounds similarly devoid of that element; and that the two classes of compounds did not combine together. He believed that this general law would elucidate other chemical doctrines, and might prove valuable in the prosecution of still more recondite principles. But a mind of such soaring aspirations was not likely to confine itself even to such a comparatively wide field as chemistry. The vast domain of medicine was before our student, rich in unexplored regions, abounding in all that could excite his eager spirit of inquiry, and reward his love of definite results. It was exactly at this period in the history of modern medicine that physicians were taking stock, as it were, of their old principles. Morbid anatomy, pursued in close connection with clinical medicine, was showing the effects of diagnosis. With the sagacious eye of one who was capable of seeing that the great necessity of the day was a science of diagnosis, Dr. Marshall Hall threw himself into the prosecution of this immensely important department of medicine at once. Here again we find fresh evidence of his eminently progressive spirit. No mere systematizing of what other men had gathered, but an original and comprehensive treatise resulted from the labors of his student life and early years in the profession.

In 1812 Marshall Hall took his degree of M.D., and shortly afterwards was appointed to the much-coveted post of house-physician, under Drs. Hamilton and Spens, at the Royal Infirmary of Edinburgh. In the following year we find Dr. Hall lecturing on the Principles of Diagnosis to a class, amongst whom were Dr. Robert Lee and Professor Grant. It was from this course of lectures that the treatise on Diagnosis, which was first published in 1817, took its origin.

In 1814 Dr. Marshall Hall left Edinburgh, after a residence there of five years. Great as was the individuality of this remarkable man, we cannot but point out that he was reared in a great school, taught by great men, and infected with an enthusiasm which prevaded, in some degree, all who came within its magical circle. Before entering upon his career as a private practitioner, Dr. Hall determined to visit some of the continental schools. We find him, therefore, shortly after his departure, successively at Paris, Berlin, and Gottingen. The journey was made partly on foot, and armed. At Gottingen Dr. Hall became acquainted with Blumenbach.

In 1815, Dr. Marshall Hall settled at Nottingham as a physician, and he speedily acquired no small reputation and practice. After a time, the appointment of physician to the General Hospital there was conferred upon him, and in that sphere he labored until his removal to London, about ten years after his first settlement at Nottingham. Of his work on Diagnosis it is almost unnecessary for us now to speak in terms of praise. Comprehensive, lucid, exact, and reliable, this work has, in the main, stood the test of forty years' trial. A better has not been produced. It was at this period of his career, too, that Dr. Hall made his researches into the effects of the loss of blood, the result of which was embodied in a paper read before the Royal Medical and Chirurgical Society in 1824. This paper and another in 1832, detailing Dr. Hall's "Experiments on the loss of blood," were published in the "Transactions of the Royal Medical and Chirurgical Society." It is hardly possible to overrate the importance of these inquiries. They revolutionized the whole practice of medicine. A new light broke in upon the medical world. A distinction, not recognized before, was drawn between inflammation and irritation. It was pointed out that delirium and excitement were by no means necessarily declaratory of cerebral or meningeal inflammation, or even congestion. Loss of blood was shown to be at the root of much that had passed before for various grades of inflammation. Practical rules were educed both for treatment and diagnosis. It was shown that active inflammation produced a tolerance of bleeding from a free opening in the upright posture; and the rare merit of supplying at once a rule of treatment and a rule of diagnosis was Dr. Marshall Hall's. Other works came forth from his pen about this time, for his mind was teeming with ideas, and his activity as an observer was unparalleled. It is hardly possible to enumerate all, but in 1827 came the "Commentaries upon various Diseases peculiar to Females"—a work which may still be consulted with advantage.

It was in 1826 that Dr. Marshall Hall sought this great metropolis as the umbilicus of the world. So active and earnest a mind could not find enough to satisfy its eager cravings in a provincial town. It was here, in this mighty city, that he determined to measure himself with numerous competitors, and to win, if possible, all the honor and all the rewards that fortune can give to those who woo her stoutly. The mind of this great man was essentially metropolitan and liberal. A fair field and no favor, and victory to the strongest, were the characteristics of his mind.

The next onward step in Dr. Marshall Hall's career was a series of researches into the circulation of the blood in the minute vessels of the batrachia. A great step in physiology resulted from these. It was shown that the capillary vessels, properly so called, are distinct, absolutely, both in structure and function, from the smallest arteries or veins; that the capillaries, or *methæmata*, are the vessels in which the nutritive changes in the economy are carried on.

But the great source of Dr. Marshall Hall's honor, the basis upon which his fame must rest in all time to come, was yet undeveloped; his paramount claims to the admiration of his cotemporaries and of posterity consists in his discoveries concerning the nervous system. Like all really important discoveries in natural science, those of Dr. Marshall Hall have had great practical effects. The soundest theory has been shown to be the best foundation for practice. That stupid heresy, that there is a vital distinction between the practical and theoretical man, was never more completely disproved than in the case of Marshall Hall. But we must endeavor to trace the progress of his researches. While engaged on the Essay on the Circulation of the Blood, it happened that a triton was decapitated. The headless body was divided into three portions: one consisted of the anterior extremities, another of the posterior, and a third of the tail. On irritating the last with a probe, it moved and coiled upwards; and similar phenomena occurred with the other segments of the body. Here, then, was a great question. Whence came that motor power? To set at rest that question, to solve that problem, has been the great labor of Dr. Marshall Hall's life.

The establishment of the reflex functions of this spinal cord; in short, the whole of the excito-motor physiology of the nervous system is the sole work of Dr. Marshall Hall. And not only this, but he has shown that there are in reality THREE great classes into which the various parts of the nervous system resolve themselves; the cerebral, or sentient voluntary; the true spinal, or excito-motor; and the ganglionic. This was the real unravelling of that perplexed and tangled web which none had before been able to accomplish. The true idea of a nervous centre could never be said to have existed before the time of Marshall Hall. The ideas of centric and eccentric action, of reflection, &c., so necessary to the comprehension of nerve-physiology, were unknown before the labors of this great discoverer. But these physiological discoveries were not mere barren facts. How rich a practical fund of therapeutical measures naturally follows the physiology and pathology of the excito-motor system, every well informed physician can testify. To departments of medical practice have gained incalculably. The success of Dr. Marshall Hall in the treatment of nervous iseases was almost entirely the result of a rigid application of his own physiological discoveries to their pathology and therapeutics. Obstetricians have found their art elevated more than any other branch of medicine. In the place of uncertain and empirical rules, there are now definite and scientific principles upon which to fall back, with the unhesitating assurance that they will stand in good stead. The most complicated of all physiological acts, viz : the act of parturition, has, by the aid of the excito-motor system, been unravelled and reduced to beautiful harmony, if not simplicity. In like manner, many of the diseases of pregnancy are explained and illuminated by the same physiological knowledge. Innumerable symptoms of other diseases are rendered intelligible and rational, which before were obscure and empirical. But to follow out the influence of Dr. Marshall Hall's discoveries through their numerous and important ramifications would be almost to write a volume on the principles of medicine. It is impossible to say when we shall cease to find some new and important application of his discoveries to the great art of healing.

We cannot pass by this period of Dr. Marshall Hall's life without remarking upon the disgraceful treatment he received from the Royal Society. The days of persecution had happily passed by, but the day of dull obstructiveness still remained. The Royal Society thought Dr. Hall's memoir "On the true Spinal Marrow and the Excito-Motor System of Nerves" unworthy of publication ! So much for the acumen of this Society. A very different verdict has, however, been given since by the great body of scientific men ; and the Society, which formerly received this great man's contribution coldly, now mourns the loss of its brightest and most illustrious member.

Since the promulgation of his researches upon the nervous system, Dr. Marshall Hall has been principally occupied with extending, applying, and developing them in every possible direction. The admirable success with which he indoctrinated the profession at large with his views must be attributed as well to his native lucidity as to their inherent truth.

During the time of Palmer's trial, it occurred to Dr. Hall to institute a physiological test for the recognition of styrchnia. As if to show the absolute correctness of his views, and how unlimited were the number and nature of the scrutinies they would bear, he found that a frog, immersed in water containing the $\frac{1}{5000}$ part of a grain of styrchnia, would, in process of time, be thrown into tetanic convulsions. For the details of these experiments we must refer to *The Lancet* of last year. The physiological test was found to be far more delicate than the chemical. Here was an instance of sagacity and precision of thought which would have done credit to any man in the flower of his age.

The last and crowning effort of Dr. Marshall Hall in the cause of science and humanity has been his discovery of what is now universally known as the "Marshall Hall Method" of restoring asphyxiated persons. How completely and irrefragably he has proved the inutility and danger of the practices hitherto in vogue for the resuscitation of asphyxiated persons! Space prevents us from going into the theoretical details of Dr. Marshall Hall's method; but our columns have, for any time these last six months, contained overwhelming proofs of its *truth* and adaptation to practice. It is pleasing to find that this last labor of a great mind has been a labor of love, something added to the stock of human happiness, something taken away from the bitterness of human life. It is singular enough that in the very place where Marshall Hall has drawn his last breath, two cases have lately occurred illustrating the superiority of the "Marshall Hall Method" over the empirical rules of the Royal Humane Society. In one case of drowning the warm bath was administered; in another, the "Marshall Hall Method" was resorted to: in the first case death was the result; in the second, restoration to life. It is also remarkable that in this number of our journal should be recorded three more examples, illustrative of the successful application of the "Marshall Hall Method" of treatment. It is curious, too, that one of them should have occurred at Nottingham.

In the practice of his profession, Dr. Marshall Hall was very successful. He linked himself early and resolutely to a great subject, and rose into fame upon his development of it. He realized an ample fortune as the reward of a life of unremitting toil. We do not mean to imply that competency was hardly earned under such conditions. Such a man would have been less than happy in a different sphere. Labor was to his restless and indomitable spirit a necessity. Even now, when we are recording the death of this illustrious and lamented physician, there is a volumn in the press,—a recent effort of his prolific mind; and until within two months before his dissolution, the mental energies of this extraordinary man were engaged in preparing for publication, in *The Lancet*, a series of papers, entitled, "The Complete Physiology of the Nervous System."

It is somewhat remarkable that Dr. Marshall Hall never held the office of physician in a hospital in London. He was only physician to a dispensary for a short time. He lectured at the Aldersgate-street and Webb-street School of Medicine, and also at St. Thomas's Hospital Medical School. He was a candidate for the Professorship of Medicine at University College upon one occasion; but owing, it is believed, to some improper influences, matters assumed such an aspect as to induce Dr. Hall to retire from the field.

We have thus far considered Dr. Hall as a man of science. In other relations of life he was equally deserving of our highest respect. As a politician, he was liberal in the highest degree. He was a strictly moral man, and was deeply imbued with a sense of the obligation of a *practical* cultivation of religion. That which he thought right to do, he *did*, with unswerving honesty and courage. All subterfuge, trickery, quackery, and guile, were utterly foreign to his nature. So simple and childlike was he in disposition, as hardly to be able to imagine in others the guile which had no home in his own breast. He was a kind husband, a most indulgent father, and a faithful friend. He married, in 1829, Charlotte, second daughter of Valentine Green, Esq., of Normanton-le-Heath, Leicestershire. Mrs. Marshall Hall's maternal grandfather was M. P. for Shaftesbury, and son of Dr. Cromwell Mortimer, physician to the Prince of Wales, father of George III. Throughout the protracted illness of Dr. Marshall Hall, the assiduous, devoted, and unremitting attentions of an affectionate wife were probably never surpassed. This testimony is due from personal observation of the fact. The deceased has left one son, who has relinquished the profession for the rural life of a country gentleman.

We must now close our notice of one over whose memory we would fain linger. Melancholy as it is to say he *was* amongst us, our sorrow is stayed by the reflection that he did not live in vain. All that a grateful profession has to give to his memory will be given. We shall still think of him with affectionate respect as a Father in Medicine, but as a child in the purity and simplicity of his mind. Though no title has adorned the name of the great Marshall Hall, we who are left behind will esteem him as one who would have graced rather than have been graced by honors however exalted. The title, which he preferred beyond all others was that of the English physiologist.

The mortal remains of this distinguished man were, on Wednesday last, removed from Brighton to Nottingham, where, we believe, a post-mortem examination has been made by his brother-in law, Mr. Higginbottom, his nephew, Mr. Higginbottom, Jr., and Dr. Ransom, physician to the Nottingham General Hospital. It is believed that the death of Dr. Marshall Hall was caused by exhaustion, produced by a stricture of the œsophagus of many years' standing, accompanied latterly, it was considered by many eminent physicians, with malignant ulceration of the part. Dr. Alfred Hall, of the old Steyne, Brighton, was one of the chief medical attendants of the deceased in his last illness. Sir Benjamin Brodie had long since pronounced the malady from which Dr. Marshall Hall was suffering to afford no hope of the application of any permanent remedy.

